# Exploring patient experiences of cancer care in Northern Ireland: A thematic analysis of free-text responses to the 2018 Northern Ireland Patient Experience Survey (NICPES)

**DOI:** 10.1186/s12913-021-06577-z

**Published:** 2021-06-07

**Authors:** Gillian Prue, Dominic O’Connor, Malcolm Brown, Olinda Santin

**Affiliations:** 1 School of Nursing & Midwifery, Queen’s University Belfast, Northern Ireland Belfast, United Kingdom; 2 School of Health Sciences, University of Nottingham, Nottingham, United Kingdom

**Keywords:** Cancer, Patient experience, Survey, Northern Ireland, Thematic analysis

## Abstract

**Background:**

Cancer diagnosis, treatment and survivorship is multifaceted, and the cancer patient experience can serve as a key indicator of healthcare performance and quality. The purpose of this paper was to analyse free-text responses from the second Northern Ireland Cancer Patient Experience Survey (NICPES) in 2018, to understand experiences of care, emerging themes and identify areas for improvement.

**Methods:**

A 72-item questionnaire (relating to clinical care experience, socio-demographics and 3 free-text questions) was distributed to all Health & Social Care Northern Ireland patients that met the inclusion criteria (≥ 16 years old; confirmed primary diagnosis of cancer and discharged between 1st May and 31st October 2017) in June 2018. Participants could complete the questionnaire online or access a free telephone support line if required. Open-ended free text responses were analysed thematically to identify common themes. Free text responses were divided into positive or negative comments.

**Results:**

In total, 3,748 people responded to the survey, with 2,416 leaving at least one free text comment (69 %). Women aged 55–74 years were most likely to comment. Overall, 3,644 comments were left across the three comments boxes, which were categorised as either positive (2,462 comments; 68 %) or negative / area for improvement (1,182 comments; 32 %). Analysis of free text responses identified six common themes (staff; speed [diagnosis and treatment]; safety; system; support services and specific concerns), which were all related to the overarching theme of survival. Staff was the largest single theme (1,458 responses) with overwhelmingly positive comments (1,322 responses; 91 %), whilst safety (296 negative comments; 70 %) and system (340 negative comments; 81 %) were predominantly negative. Negative comments relating to primary care, aftercare and the cancer system were reported.

**Conclusions:**

The high response rate to the free text comments indicates patients were motivated to engage. Analysis indicates most comments provided were positive in nature. Most survey respondents reported a positive experience in relation to staff. However, there were a number of areas for improvement including the aftercare experience, and a perceived disconnect between primary care and cancer services. These results can help inform the effective delivery of cancer services in Northern Ireland.

## Background

Cancer remains a leading cause of mortality worldwide, with the current lifetime risk of developing cancer estimated at 1 in 2, for those born after 1960 [1]. Each year over 360,000 new cancer cases are reported in the United Kingdom (UK), adding to the estimated 2.9 million individuals currently living with the disease [2]. In 2018, 14,039 individuals living in Northern Ireland were diagnosed with cancer [3]. With increasing survival rates, more people are living longer with and beyond their cancer. For many of these individuals, the cancer journey through diagnosis, treatment and beyond can be a long and repetitive process, involving several stages of investigation, treatment, and multiple encounters with a variety of health professionals and services. Throughout this journey, focus is placed on delivering high quality health care, and there is an increasing recognition of patient perception as a key indicator of quality of care [4]. Assessing the cancer patient experience of care can provide a rich and valuable insight into healthcare performance, and how the healthcare system impacts on the patients experience throughout their cancer journey. These insights can help highlight areas which work well and help identify areas for improvement, with the outcome of patient surveys leading to quality improvements in both American and European healthcare settings [5].

Within the UK, there has been an increasing emphasis on understanding and improving the cancer patient experience, with several national surveys conducted to gather information on healthcare performance. The National Cancer Patient Experience Survey (CPES) is an annual survey which began in England in 2010 [6], and has also been completed in both Scotland [7], and Wales [8]. The Public Health Agency (PHA) in Northern Ireland introduced the Northern Ireland Cancer Patient Experience Survey (NICPES) in 2015. The 2015 survey highlighted that the majority of cancer patients report a positive experience of care. However, areas for improvement were highlighted, including access to a Clinical Nurse Specialist (CNS) and the provision of information [9]. In 2018, Macmillan Cancer Support, the Northern Ireland Health and Social Care Board and the Northern Ireland Cancer Network (NICaN) worked in partnership to deliver the second NICPES [10]. The aim of this survey was to again provide further reliable measurement of cancer patients’ experiences of care in Northern Ireland and to identify areas for improvement.

The majority of the information collected through the NICPES 2015 and 2018 was in the form of categorical survey response data; however, such quantitative data may provide insufficient detail to facilitate appropriate changes [11]. Previous UK surveys have included opportunities for free text comments. The inclusion of such questions provides patients with the opportunity to give detailed and anonymous feedback on their experience of care. The collection of this qualitative patient experience data can give depth and context to quantitative findings, thus providing a greater understanding of aspects of cancer care that are working well and those that require further improvement [12]. In addition, the inclusion of such questions allows for comparisons between regional patient experience surveys, which have included similar free text questions and enables local monitoring of progress on cancer care, providing evidence that can be used to drive quality improvements. The analysis of free text responses to the CPES from patients in London Trusts [13], Wales [8] and Scotland [7] has previously been undertaken. As such, including free text questions at the end of the NICPES 2018 survey was approved, providing an opportunity for patients to comment on their cancer care in more detail.

## Methods

### Study design and participants

This study involved analysis of responses to open ended survey questions collected during the 2018 NICPES service evaluation. The sample included all Health & Social Care Northern Ireland patients who met the following inclusion criteria: ≥ 16 years old; had a confirmed primary diagnosis of cancer with an International Classification of Disease (ICD10) code of C00-C99 or D05; discharged from a hospital within the Trust (inpatient or day case) between 1st May and 31st October 2017. Results from the NICPES 2018 closed questions were published in January 2019 [10] with a response rate of 57 % (*n* = 3,748).

### Survey content

The questionnaire included 62 questions covering aspects of the clinical care experience, and 7 questions regarding sociodemographic [10]. After the closed questions, participants were invited to include comments on three free text questions:

1. Was there anything particularly good about your cancer care in Northern Ireland?
2. Was there anything that could have been improved?
3. Do you have any other comments that you wish to make?

### Survey process

The survey (including cover letter, questionnaire, information sheet, and consent) was distributed by post in June 2018, with two reminders sent to non-responders in July 2018. Survey packs included an option to complete online, and details for a free telephone line which patients could call to ask questions, complete the survey verbally, or to access an interpreting service [10]. All personal information was handled securely in line with the General Data Protection Regulation and Data Protection Act (2018). By completing the questionnaire, participants gave their consent for the information they provided to be used for the purposes specified within the information sheet.

### Analysis

The free text comments were analysed thematically according to Miles and Huberman’ techniques of labelling, coding, categorising and theme development [14]. The process involved identifying commonalities in the dataset and searching and comparing the free text responses to identify relationships and themes. All free text responses were read by OS and GP, with codes (themes/subthemes) applied to all responses. A coding framework was developed iteratively (by GP and OS) through reading, coding and discussing the texts of the first 10 % of responses. The level of agreement between the researchers regarding coding categories and potential themes was assessed and discussed, ensuring that the category given to a section of data was fitting. Responses were searched for data that may have contradicted the emerging themes. This process continued until agreement was greater than 80 %. Constant comparative techniques were used to ensure all perspectives were represented in the analysis, and deviant cases examined. At each stage, findings were verified and discussed by the research team in order to assess accuracy and credibility of the interpretation, promote inter-rater reliability and ensure rigour [15]. To understand patterns and differences dependent on patient demographics, the team adopted a similar approach to analysis outlined in the Scottish Cancer Experience Survey 2015/16 [7]. The proportion of participants who made a positive comment were compared with the proportion that made a negative comment across each demographic category, for each of the identified themes.

Each free text box contained a variety of comments across a number of themes. Therefore, positive and negative comments have been presented under each theme as per the first two comments boxes (*was there anything particularly good about your cancer care in Northern Ireland; was there anything that could have been improved?).* For comment box 3 (*do you have any other comments that you wish to make?)* dependent on the content (i.e. whether it was a positive or a negative comment) these have been included in the respective positive and/or negative section. The report uses verbatim comments to illustrate the themes, but any identifiable data has been removed.

### Ethical approval

 Approval for the analysis of the fully anonymised comments by the research team was given by the Health and Social Care Board Information Governance Committee. Participants provided consent for use and publication of anonymised data.

## Results

Of the 3,748 respondents to the survey, 2,416 (69 %) left at least one free text comment. Women aged between 65 and 74 yrs. who were retired were more likely to leave a comment, whilst those aged 65–74 yrs., from the most deprived areas, with a breast or haematological cancer diagnosis were less likely to leave a comment. The socio-demographic and clinical characteristics of all respondents and those who left at least one comment are summarised in Table 1.

**Table 1:** Socio-demographicandclinicaldataof all respondents and those who left at least 1 comment.

	**All respondents**		**Respondents with at least 1 comment **	
**Respondent characteristics**	**Number of respondents**	**Percentage**	**Number of respondents**	**Percentage**
**Sex**				
Male	1639	47.1%	1121	46.6%
Female	1839	52.9%	1284	53.4%
**Age group**				
< 45	92	2.7%	68	2.9%
45 - 54	312	9.0%	234	9.7%
55 - 64	809	23.3%	555	23.1%
65 - 74	1039	29.9%	732	30.4%
75 - 84	548	15.8%	365	15.2%
85 +	31	0.9%	19	0.8%
**Ethnicity**				
White	3220	99.5%	2239	99.6%
Any other ethnic group	16	0.5%	8	0.4%
**Sexual orientation**				
Heterosexual or straight	3119	96.6%	2186	97.2%
Gay, Lesbian, Bisexual, Other	41	1.3%	23	1.0%
Prefer not to say	59	1.8%	40	1.8%
**Deprivation quintile**				
1 (Least deprived)	613	17.6%	401	16.7%
2	667	19.2%	452	18.8%
3	667	19.2%	453	18.8%
4	728	20.9%	498	20.7%
5 (Most deprived)	803	23.1%	601	25.0%
**Cancer type**				
Brain / CNS	29	0.8%	21	0.9%
Breast	655	18.8%	483	20.1%
Colorectal / LGT	431	12.4%	290	12.1%
Gynaecological	182	5.2%	127	5.3%
Haematological	622	17.9%	432	18.0%
Head and Neck	109	3.1%	77	3.2%
Lung	180	5.2%	122	5.1%
Prostate	301	8.7%	211	8.8%
Sarcoma	18	0.5%	13	0.5%
Skin	68	2.0%	48	2.0%
Upper Gastro	152	4.4%	101	4.2%
Urological	370	10.6%	230	9.6%
Other	361	10.4%	250	10.4%
**Health Board**				
Belfast	1455	41.8%	1052	43.7%
Northern	424	12.2%	303	12.6%
South Eastern	598	17.2%	423	17.6%
Southern	473	13.6%	284	11.8%
Western	528	15.2%	343	14.3%
**Employment status when diagnosed with cancer**				
Employed (FT/PT)	1243	37.5%	909	39.6%
Unemployed	173	5.2%	106	4.6%
Retired	1624	49%	1110	48.4%
Other (incl. homemaker, student)	272	8.2%	169	7.4%
**Employment status now**				
Employed (FT/PT)	686	21.0%	511	22.6%
Unemployed	410	12.6%	262	11.6%
Retired	1908	58.5%	1326	58.6%
Other (incl. homemaker, student)	260	8.0%	165	7.3%

**Fig. 1:**
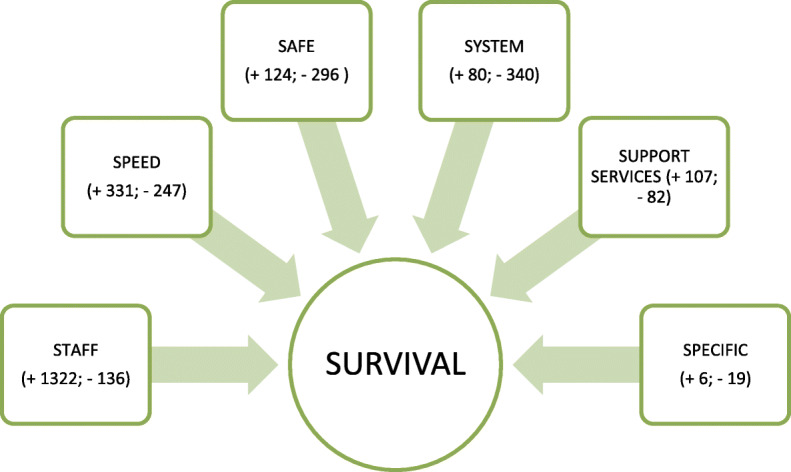
Themes identified in free text responses (+ denotes positive, - indicates negative)

Overall, 3644 comments were left across the three comments boxes, which could be categorised as either a positive or negative comment. There were 2,462 comments that were perceived to be of a positive nature and 1182 comments focused on issues that could be improved. Positive comments accounted for 68 % of total comments. Analysis of the free text responses identified six common themes which were all related to the overarching theme of *survival* (see Fig. 1).

1. Staff.
2. Speed (of diagnosis and treatment).
3. Safe.
4. System.
5. Support services.
6. Specific concerns.

### Survival

Having a diagnosis of cancer and the worry and uncertainty associated with this,as well asthe fear of not surviving was embedded throughoutallthe positive and negative comments. Participants wanted staff they couldtrust,that they felt safe within terms of their ability tocare for them professionallyand effectively,and expert clinicians who coulddiagnose and treatthemquickly in an expert centre.Patientsalso commentedon the support they received via various voluntary agencies,and the positive (and sometimes negative) impact this had on their experience. Somepatientsalso had very specific concerns surrounding their cancer experience, whichagain often negatively impacted on their survival belief.

### Staff

The largest single theme was staff (1,458 responses), which was overwhelmingly positive with an approximate ratio of 10:1 positive to negative comments. Most positive responses focused on how staff of all levels positively impacted on the cancer experience throughout cancer diagnosis, treatment and care.

#### Staff attitude to patients

Participants commonly reported that cancer staff treated them with professionalism and kindness which greatly improved their experience and enabled them to cope.Respondents felt welcomed and supported by all staff groups and grades,including medical, administrative, cleaning and hospitality staff. This led to an overall sense of support and kindness within the cancer care setting.

*“I feel my care has been second to none, from the porters, nurses, doctors, specialists and all other department specialists and all other department specialists i.e. scan, X-ray etc. I have been treated with respect and dignity. Everyone has been a pleasure to meet, including the admin staff during my treatment”*(65 - 74 years, Haematological cancer).

*“The staff from consultants to nurses to administration to cleaners have all been amazing and they've all made having cancer bearable” *(65 - 74 years, Haematological cancer).

In addition to the warmness and kindness demonstrated by staff, patients acknowledged the good communication skills of staff and the positive influence this had on patient experience. The communication style of the health care professionals improved patient experience as patients felt that they were involved in their treatment and care decisions. Many patients reported that medical staff helped them to understand their diagnosis and that they could ask questions. Importantly they felt like a person and not simply a patient. Respondents noted a high level of gratitude, particularly related to the view that staff had saved their lives and made a difficult process bearable.

*“Despite the fact that there are so many cancer patients to deal with, all the doctors and nurses with whom I came into contact made me feel that I was being cared for in an individual way. They made time to answer my questions***”**(65 - 74 years, Breast cancer).

In contrast to the majority of patients reporting good communication a small proportion (10%) noted that they experienced some staff as abrupt which left them feeling disempowered:

*“I understand that the consultants are the experts, but when it comes to decisions regarding getting chemotherapy and radiotherapy, I feel that the patient and family should have a bigger say”*(65 - 74 years, Lung cancer).

*“After 12 days on the ward I was told on the morning I was due to be discharged that results showed I had cancer. This was given to me by my surgeon on the ward while surrounded by nurses and others (not sure who all were now). Feel this could have happened more privately as this came as a shock to me”*(55 - 64 years, Other cancer).

There was recognition that staff were busy, with many commenting on perceived staff shortages, but patients were very thankful for how despite this, staff continued to approach and treat them with care, kindness and dignity.

*“Although very short staffed the nurses and doctors still have time for you, reassuring me of all my worries. Always make me feel at ease” *(65 - 74 years, Haematological cancer).

*“I owe my life to a huge team of doctors, nurses, surgeons and many other working in the National Health Service. It is a debt that I will never be able to repay” *(65 - 74 years, Colorectal cancer).

*“I am extremely grateful to all staff. You are all angels on earth” *(45 - 54 years, Breast cancer).

In contrast a poor attitude from staff was reported,particularly as inpatients on general wards. This was described as some staff having little empathy or understanding of the patient’s cancer conditions, needs or sensitivities and resulted in patients feeling like they were not cared for as an individual(in direct contrast to the positive experiences described above).

*“Ward nurses need to be a little more compassionate and understanding. Aware of surgery conditions, not treat patients like being on a conveyor belt!”*(45 - 54 years, Breast cancer).

### Speed

Speed of diagnosis and associated treatment was commonly discussed by participants. A fast diagnosis and treatment, and conversely a perceived delay in diagnosis and treatment, greatly influenced their experience and their perception of their chances of treatment being effective and thus surviving their cancer.

Positive experiences discussed included the perceived speed of their diagnosis and/or how quickly they received their treatment (surgery, radiotherapy, chemotherapy) once in the cancer care system. The positive attitude to this appeared to be linked to the belief that this would enhance their chances of surviving their cancer and their treatment being more effective. Diagnosis and treatment were described by many as co-ordinated and progressed at a satisfactory pace, with many describing the service as a one-stop shop.

*“I was referred really quickly by my GP [General Practitioner] and seen by a specialist quite quickly. My operation happened quickly, which meant that my cancer had less time to spread” *(45 - 54 years, Skin cancer).

*“I would have to agree that once my diagnosis was made and I got "into the system", all care for me could not have been better” *(65 - 74 years, Haematological cancer).

*“Got very speedy treatment after diagnosis. Everything moved along smoothly and seemed to be well coordinated. Oncologists and surgeon seemed to work well together” *(65 - 74 years, Colorectal cancer).

*“Prompt 'one stop shop' for assessment, scans, biopsy and diagnosis. All on one day” *(35 - 44 years, Breast cancer).

However, despite this positive experience of speed of diagnosis and treatment with the cancer system, a number of negative experiences in relation to speed of diagnosis were discussed at a primary care level. Some participants described making several visits to their GP before they were referred to oncology for further investigations. Comments indicated that this caused distress, particularly as it led to a belief that their disease may have progressed prior to diagnosis or commencing cancer treatment. Some respondents reported having to take the private healthcare route to speed up the process and avoid longer delays, further highlighting the importance and significance of time and speed to potential cancer patients.

*“Once I got to the hospital the speed and efficiency was excellent but getting the GP to give a proper diagnosis was dreadful and she should be ashamed of herself for the lengthy delay. This could have cost me my life” *(55 - 64 years, Sarcoma).

*“I had to go private because it would have been four weeks before I would have been seen by a specialist. I feel that's not good enough. My consultant told me I had done the right thing by going private, because the type of cancer I had was very aggressive” *(45 - 54 years, Breast cancer).

*“The delay from diagnosis to surgery was delayed by weeks, due to Easter holidays, consultant holidays and then May Day holiday” *(55 - 64 years, Breast cancer).

### Support services

Commonly, participants discussed the positive support and care they received from support and voluntary services such as, exercise classes, yoga, wig fitting and make-up services; tea and coffee provision at clinics; and complementary therapy. These services appeared to help patients with self-efficacy, self-esteem and confidence.

*“Macmillan support was fantastic, especially the 'Move More Programme'. I think medical and nursing staff should highlight 'Move More' and really encourage patients to take part”. *(45 - 54 years, Breast cancer).

*“I also was able to use some of the Macmillan services, e.g. yoga, which were very helpful during my recovery. The feel good, look good make up service was a real treat. Also, the free wigs were unexpectedly great”*(55 - 64 years, Breast cancer).

However, it should be noted that a proportion of patients reported negative experiences in relation to the availability of additional supportive services, particularly when they perceived that these were not made available to them or that they were not informed of what was available.

*“The one thing I have noticed is that Macmillan did not offer me any services. I was told about a group that met every month but told I didn't need to go. Why? I don't know. I also signed up for exercise classes, but no-one ever contacted me, even though they said they would. I feel that because of the cancer I have they wanted to keep me away from other cancer sufferers who had more hope. But I need help too. I did receive financial help which was great” *(45 - 54 years, Lung cancer).

There is also a need to further promote financial support for someone undergoing cancer treatment in the future.

*“I would like to know more about financial benefits available to me” *(35 - 44 years, Colorectal cancer).

### Safe

A consistent theme across responses was the perception that staff were competent, skilled and professional, and that there were available measures in place to monitor patients’ well-being. These aspects tended to reduce patients fear of recurrence, improved their perceptions of their chances of survival and made them feel safe. This theme focuses on how patients felt reassured as they were monitored, and they were able to contact the correct professional when any worrying issues arose. Under the theme Safe, three subthemes were identified: Cancer fear / safety, Illness / symptom monitoring and Aftercare.

#### Cancer fear/safety

Respondents reported that staff were experts who they trusted and felt safe with, which reduced the fear that surrounds a cancer diagnosis. Patients reported that they perceived the cancer staff to have a high level of technical and medical skills, and expertise to advise on the best course of treatment, provide the treatment and manage the patient’s cancer. Again, these comments related to various healthcare professionals, from their surgeon/oncologist to their GP, CNS / nurse and radiographer.

*“Northern Ireland has an amazing high standard of excellence in regard to cancer care. We are so fortunate to have such expertise available” *(55 - 64 years, Other cancer).

CNS in particular were cited as having a positive impact around reducing the fear of a cancer diagnosis. They were described as cancer experts who were supportive at time of diagnosis, very knowledgeable and approachable. Many patients described the CNS as a steady and constant figure throughout all stages of the cancer journey and that this continuity helped them to build a relationship and feel safe.

*“Having a nurse specialist, we would have been lost without her. She was always there to explain what was happening and keep us positive” *(65 - 74 years, Lung cancer).

#### Illness/Symptom Monitoring

Patients described how they perceived that they were constantly monitored (for signs of recurrence or progression). The knowledge that there was a helpline, the red flag system, or a CNS being available and contactable made a positive difference to how they felt towards their diagnosis and treatment. The perception that they could receive immediate help and support if they experienced any problems was reassuring. Having these means of contact also meant any issues would be picked up quickly and could be dealt with before their illness or condition progressed further.

*“Chemotherapy staff/nurses were an amazing support. So caring and helpful… Any issues, they were just a phone call away”* (35 - 44 years, Breast cancer).

*“Access to cancer professionals outside of normal business hours excellent, a type of safety net*” (65 - 74 years, Gynaecological cancer).

#### Aftercare

Commonly patients reported negative experiences of aftercare particularly in relation to symptom management, unclear follow up plans, and delays with follow up appointments. These issues exacerbated their fear that their disease may progress or recur undetected.

On completion of treatment, respondents frequently reported feeling abandoned and not knowing what to expect next with their care. This caused a degree of distress and anxiety. This refers to the issue of trusting knowledgeable staff, as without this contact, patients felt less prepared to cope and manage. They also described lacking the confidence to contact their oncologist or CNS after their treatment was finished, as they perceived they were no longer the priority for the staff in the cancer treatment centres. Patients reported feeling uneasy accessing their GP for help and support due to concerns that the GP would not have a comprehensive understanding of their illness and be unable to treat late effects of treatment or spot recurrence. Participants indicated that a lack of communication between cancer services and the GP added to this issue.

*“The care while receiving treatment was excellent, but since my appointment and treatment stopped, I've had no contact with anyone in the form of aftercare. I feel this is what lets the HC [healthcare] system fail. Sometimes I feel like I've been left to manage with all my worries on my own *(45 - 54 years, Breast cancer).

*“After treatment care, not explained what happens next with my cancer. Don't know if it's clear or will be coming back. Just in limbo” *(85+ years, Lung cancer).

*“My GP despite being kept informed by the hospital seems fairly ignorant about my condition and treatment. As a result of this my first port of call tends to be the hospital helpline, rather than the GP. Felt less at risk of infection in haematology. Also, the staff were fully aware of my condition and the treatment I needed” *(55 - 64 years, Haematological cancer).

*“Communication between cancer doctors and GP's could be improved. Very long delay in my GP being made aware of changes and updates to my medication and care” *(75 - 84 years, Prostate cancer).

*“The follow up care is poor and very far apart, no feedback from any appointment and requests for scans were ignored, symptoms worsened and it was only after 2 visits by ambulance to hospital that scans were eventually carried out. The outcome is that cancer has spread, and no treatment can be given” *(65 - 74 years, Colorectal cancer).

Linked to issues with aftercare, some respondents described how their treatment-related symptoms were poorly managed post treatment, and equally that the extent and severity of post-treatment symptoms were more severe than initially anticipated.

*“The oncologist could have explained about the hormone treatment, i.e. Anastrozole 1mg for the next five years, I have to take what symptoms it caused, i.e. sweats, hot flushes, joint pain, mood swings” *(65 - 74 years, Breast cancer).

### System

#### Clinic delays

Comments regarding the set up and co-ordination of the cancer system following diagnosis were overwhelmingly negative. A commonly occurring sub-theme focused on the environment in which treatments were delivered. Respondents described overcrowded clinics with a lack of available seating leading to some people having to sit on the floor. Overcrowded clinics, long waits and lack of seats when going through a difficult and emotional time were described as adding to the burden of treatment days with many patients reporting that this exacerbated the feeling of exhaustion.

*“I started my third chemotherapy just yesterday. The clinic was so busy yesterday the two waiting rooms were full. Some patients sitting on the floor. I appreciate this was an exceptionally big clinic, which is not fair on the staff”. *(65 - 74 years, Other cancer).

Comments described the difficulty of having to attend the chemotherapy clinic early in the morning for an appointment, having bloods taken and then having to wait for most of the day for pharmacy to *‘make up’* their chemotherapy to take home. Many patients lived far from the clinic and had travelled long distances to the clinic and home again, making for a very long and tiring day for many (particularly with the described clinic delays).

*“I arrived for my 9:30am appointment at 9:15am and left the hospital at 5pm. There was a three-hour delay in pharmacy. This is really tough for both patients, doctors and nursing staff” *(65 - 74 years, Other cancer).

It should be stressed that, although waiting for long periods in a crowded area was a frequently recurring experience, many patients caveated their response with stating that it was understandable and bearable, that they greatly appreciated how busy the staff were and that they were clearly doing everything they could to help in a difficult situation. For many, it would appear that, they were including the comment reluctantly and did not want it to detract from their overall very positive cancer experience.

*“I won't complain about waiting times at weekly (Monday to Thursday) clinics to see consultant and have treatment. I am just grateful that I am having treatment and by such wonderful nurses and doctors in my opinion we (the cancer patients) are all in the one boat. The clinic is busy! The doctors and nurses and staff are all working flat out. They are doing their very best to make our treatment as comfortable and easy as possible” *(45 - 54 years, Haematological cancer).

A commonly occurring theme within the cancer system was negative experience regarding continuity of care. This centred on patients being reviewed by different oncologists and other health care professionals when they attended appointments, which appeared to reduce their confidence in their care and treatment.

*“I find dealing with different doctors every time I go to the hospital very difficult as you have to explain everything all over again and I know that on some occasions they have missed or failed to take note of some of my problems” *(65 - 74 years, Breast cancer).

#### Non-cancer system

Some comments appeared to be describing a negative experience when the patient was cared for in a non-cancer ward or when they presented at accident and emergency (A&E) with complications. When cancer patients presented or were cared for outside of the cancer services, they perceived that medical and nursing staff had a lack of skills and understanding about cancer, cancer treatments, and the potential complications that stem from treatment. These issues added to patient perceptions of feeling unsafe and not being appropriately cared for.

*“I was admitted to A&E during my treatment and found that staff there were not very aware of my condition and were not trained in how to take blood from a PICC line which meant that I was having to have cannulas/needles inserted, which was uncomfortable” *(35 - 44 years, Breast cancer).

Some patients described how the helpline aided and removed some of the difficulties outlined above. The helpline was described as a fast track way into the cancer system, with cancer specialists being ready to care for them when they experienced difficulties.

*“I found the helpline exceptionally good. I had to be hospitalised due to neutropenic sepsis. I was able to contact them direct and was told to come to the hospital where they were waiting on me rather than sitting in an A&E department, feeling so unwell - waiting for hours” *(55 - 64 years, Breast cancer).

#### Cancer-specific care

Treatment at dedicated cancer centres (the Northern Ireland Cancer Centre [NICC] and North West Cancer Centre) appeared to contribute to a positive care experience. Patients indicated that as a result of attending a cancer centre, all required services were within the same unit and staff within these units were perceived as experts.

*“I feel very fortunate to be on the 'doorstep' of the cancer centre/hospital benefiting from top care and research associated with QUB [Queen’s University Belfast]” *(55 - 64 years, Haematological cancer).

### Specific concerns

A small proportion of patients reported specific issuesrelatingto their own unique experience in hospital, for example,complications following surgery, not receivingmedications, poor toilet facilities, or issues with the quality of hospital food.

*“My stay in hospital was too short.Surgery on Wednesday,discharged on Friday. No blood thinning medication given at x. I was admittedto xa few days later with lung clots and pneumonia. Think this could have been prevented if I had more recovery time at x”*(65 - 74 years, Lungcancer).

*“Because of my surgery I had to start on pureed food, which was 'awful' to say the least. Not much to eat for someone having my type of surgery!”*(65 - 74 years, Prostatecancer).

*“The only problem I could find was that the ward toilet was only cleaned once in the morning. However, people in the ward with the problems/conditions they had sometimes left the toilets in a terrible state and subsequent users could have picked up 'infections'.”*(55 - 64 years, Upper GIcancer).

On occasion, patients with a cancer that affected their digestive system reported that the quality and types of foods provided were inappropriate, with the service not meeting their particular dietary requirements.

*“Because of my surgery I could hardly eat anything and the food that they gave me was terrible I couldn’t eat it. I think they could improve the meals they give particularly to people who have problems with their guts”*(65 - 74 years, Colorectal cancer).

## Discussion

This is the first national survey to document the patient experience of cancer care in Northern Ireland by analysing free text comments of patients. Respondents detailed issues which they felt were important or had an overall impact on their care experience. The high response rate to the free text comments (69%) suggests that patients were motivated to engage with the opportunity to provide comments on their experience of care. The analysis indicated that the majority of comments provided were of a positive nature (68%), which reflects the findings of the closed questions of the NICPES 2018 [10] and is a consistent finding with other national surveys [7, 8, 13]. However, negative comments were reported, pertaining aftercare, primary care, and the cancer system. Analysis of the closed questions in the NICPES 2018 found that patients reported an overall high rating of care (8.97, scale: 0- very poor, 10-very good) [10]. The inclusion and analysis of the free text comments provided a greater insight into the areas of care in which patients had reported on negatively.

Most survey respondents reported a positive experience in relation to the care and treatment they received from staff. Patients reported that staff of all levels had an excellent communication style. This is important, as exemplary service from frontline service staff (e.g. receptionists) can influence other subsequent interactions with clinical staff [16]. Indeed, respondents made reference to situations from the way they were greeted at reception to interactions with the CNS. Of note, the CNS had a positive impact on the patient experience through acting as a consistent source of support and closely monitoring for signs of recurrence or progression. This finding is supportive of the NICPES 2018 quantitative findings which demonstrated that the most increased score since NICPES 2015 [9] was regarding CNS provision. The percentage of respondents stating that they had been given the name of a CNS who would support them through their treatment increased from 72% in 2015 to 82% in 2018 [10]. In addition, the positive staff experience supports the quantitative findings, which demonstrated that patients reported a significant increase since NICPES 2015 in the confidence and trust in the ward nurses treating them [9]. The positive experiences in relation to staff is similarly reported in London [13] and Scotland [7]. It should be noted that a smaller proportion of respondents within the free text comments (*n*= 136) reported negative interactions with staff; however, this typically was discussed within the context of general wards, where patients described poorer interactions with staff that lacked understanding and empathy.

Analysis of the negative comments indicated a negative aftercare experience. This included unclear follow up plans and delays with receiving follow-up appointments. These experiences led to patients feeling unsafe and unsure of how to best manage their symptoms. Similar experiences of aftercare have been reported in patients in Scotland [7], and Wales [8]. The post treatment phase is commonly highlighted as a period when patients can feel ‘abandoned’ by the health care system [17]. Indeed, previous reports have suggested as many as 40% experience these feeling during this time period [18]. As such, feeling ‘cut off’ after receiving intensive support during treatment can lead to short- and long-term emotional distress [19]. Evidence suggests that although few patients have unmet psychosocial needs when treatment ends, this can increase to 50% during survivorship [20, 21] and for 60%, these issues may not resolve within 6 months [17]. There is an established recognition for increased support during survivorship, and the results of this study emphasise the need for future development in this area across Northern Ireland.

A lack of communication and co-ordination between primary care and cancer services during the cancer follow-up phase was reported by patients. GPs were perceived by patients as being unaware of changes to medications and how their cancer care should be managed. The GPs perceived lack of information regarding the patient’s management plan was reported to cause worry and anxiety. The quantitative findings demonstrated that there was a considerable drop in the proportion of patients who felt that primary care staff did everything that they could to support them while they were having their cancer treatment [10]. This is of considerable importance since primary care is generally the first port of call for many patients in the community. Potential consideration should be given to a more holistic approach, with enhanced support and earlier collaboration with patients and the involvement of GP’s [22]. Cancer services and primary care providers need to work to increase communication and partnership, to improve the treatment and follow-up care for cancer patients living in their community, which will become more pertinent in future with the increasing number of cancer survivors [23].

Patients expressed an overwhelmingly negative experience with the set-up / coordination of the cancer system. Delays in diagnosis and treatment were often reported. Such issues may lead to a certain level of inequality, with those being able to fund private healthcare potentially receiving their diagnosis and treatment faster than those who could not [24]. It should be noted that issues with delays in diagnosis and treatment were not only reported in primary care; there were also some comments on delays within secondary care (e.g. cancelled surgery; delays with diagnostic scans). Patients reported overcrowded oncology clinics, and long days due to treatment delays, both of which added to the burden and fatigue of clinic visits. Our findings indicate consequences of overcrowding which is common in UK outpatient healthcare [25], and is a key area for improvement within most healthcare settings. These findings are unsurprising given current oncology workforce shortages are estimated at 18% and are expected to increase to 22% by 2023 [26]. Indeed, patients in the current study made reference to inadequate staffing levels, echoing observations from Bracher et al. [8] and Wiseman and colleagues [13]. However, patients in the current study did communicate an understanding of this situation, and were appreciative that although staff were extremely busy, they were doing everything they could to help in a difficult situation.

There are a number of established country-specific frameworks for evaluating the quality of cancer care (e.g. the National Initiative for Cancer Care Quality (NICCQ) [27] and the Quality Oncology Practice Initiative (QOPI) [28] in the United States and the Cancer System Quality Index (CSQI) in Canada). A review of European nations also identified numerous other initiatives that developed indicators for measuring quality in cancer care [29]. Chiew et al. (2018) recently conducted a narrative synthesis of available cancer care quality assessments, with the aim of creating an integrated conceptual framework. This framework includes many of the domains assessed in the NICPES, and highlights the importance of incorporating patient experience within this, emphasising this is as crucial as disease outcomes [30]. This integrated framework also highlights the significance of including a ‘timeliness of care’ domain, which was of vital importance to the respondents in the NICPES 2018, given the impact this was perceived to have on their chances of survival.

### Study limitations

The aim of free text questions is to provide a deeper insight into patients’ experiences as quantified in numerical responses of the NICPES 2018 survey. These results however should be interpreted with caution as free text responses were often limited to one or two sentences. This restricted information, given in open-ended survey responses, reduces the potential to understand the context of the patient experience. A number of respondents did not provide free text responses. This may be indicative of participant burden in relation to survey completion or it may also be the case that these individuals experienced negative experiences and were uneasy documenting these. The issue of recall bias should also be considered when interpreting the results, as for some patients it may have been some time since they have received care from the cancer system.

## Conclusions

Generally, respondents reported a very positive experience of the cancer service in Northern Ireland. Positive experiences were attributed to the caring and professional nature of cancer staff, with emphasis given to the role of the CNS and supportive of the findings of the quantitative survey [10]. However, there were a number of areas identified for improvement, including how patients can be supported into survivorship. The need to improve primary care provision for cancer patients, and the burden and fatigue of overcrowded cancer clinics were headline findings in the qualitative findings. Finally, there is a need to improve awareness and accessibility of additional voluntary support services in the future (i.e. Macmillan Move More, Financial info, Macmillan Information and Support Services). The results of this study can help inform how cancer services can be delivered more effectively with continued patient care. 

## Data Availability

Source data for the study are free-text responses to the Northern Ireland Cancer Patient Experience Survey (NICPES) for 2018. These data are available from the survey provider (Quality Health, UK) https://www.quality-health.co.uk/ email: info@quality-health.co.uk.

## Abbreviations

NICPES: Northern Ireland cancer patient experience survey
UK: United Kingdom
CPES: Cancer patient experience survey
PHA: Public Health Agency
CNS: Clinical nurse specialist
NICaN: Northern Ireland Cancer Network
ICD: International classification of disease
GP: General practitioner
A&E: Accident and emergency
NICC: Northern Ireland Cancer Centre
QUB: Queen’s University Belfast
GI: gastrointestinal
NICCQ: National Initiative for Cancer Care Quality
QOPI: Quality Oncology Practice Initiative
CSQI: Cancer System Quality Index
GDPR: General Data Protection Regulation
